# Colorimetric tests for diagnosis of filarial infection and vector surveillance using non-instrumented nucleic acid loop-mediated isothermal amplification (NINA-LAMP)

**DOI:** 10.1371/journal.pone.0169011

**Published:** 2017-02-15

**Authors:** Catherine B. Poole, Zhiru Li, Andy Alhassan, Dylan Guelig, Steven Diesburg, Nathan A. Tanner, Yinhua Zhang, Thomas C. Evans, Paul LaBarre, Samuel Wanji, Robert A. Burton, Clotilde K. S. Carlow

**Affiliations:** 1 New England Biolabs, Ipswich, MA United States of America; 2 PATH, Seattle, Washington, United States of America; 3 Research Foundation in Tropical Diseases and Environment, Buea, Cameroon; George Washington University School of Medicine and Health Sciences, UNITED STATES

## Abstract

Accurate detection of filarial parasites in humans is essential for the implementation and evaluation of mass drug administration programs to control onchocerciasis and lymphatic filariasis. Determining the infection levels in vector populations is also important for assessing transmission, deciding when drug treatments may be terminated and for monitoring recrudescence. Immunological methods to detect infection in humans are available, however, cross-reactivity issues have been reported. Nucleic acid-based molecular assays offer high levels of specificity and sensitivity, and can be used to detect infection in both humans and vectors. In this study we developed loop-mediated isothermal amplification (LAMP) tests to detect three different filarial DNAs in human and insect samples using pH sensitive dyes for enhanced visual detection of amplification. Furthermore, reactions were performed in a portable, non-instrumented nucleic acid amplification (NINA) device that provides a stable heat source for LAMP. The efficacy of several strand displacing DNA polymerases were evaluated in combination with neutral red or phenol red dyes. Colorimetric NINA-LAMP assays targeting *Brugia Hha* I repeat, *Onchocerca volvulus GST1a* and *Wuchereria bancrofti* LDR each exhibit species-specificity and are also highly sensitive, detecting DNA equivalent to 1/10-1/5000^th^ of one microfilaria. Reaction times varied depending on whether a single copy gene (70 minutes, *O*. *volvulus*) or repetitive DNA (40 min, *B*. *malayi* and *W*. *bancrofti*) was employed as a biomarker. The NINA heater can be used to detect multiple infections simultaneously. The accuracy, simplicity and versatility of the technology suggests that colorimetric NINA-LAMP assays are ideally suited for monitoring the success of filariasis control programs.

## Introduction

Filariasis is a parasitic disease caused by any one of several tissue-dwelling, filarial nematodes. The parasites are highly prevalent in regions of Africa, Asia, South and Central America, and the Yemen peninsula [1]. In Africa alone, more than 25 million people have onchocerciasis, or ‘river blindness’ [2] and worldwide 40 million suffer from lymphatic filariasis (LF), also known as elephantiasis [3]. Onchocerciasis is caused by the subcutaneous dwelling parasite, *Onchocerca volvulus*. First stage larvae, known as microfilariae (mf), released upon mating of adult parasites, migrate throughout the skin and eyes leading to an intense itch associated with papular dermatitis as well as severe ocular damage culminating in blindness [4]. The clinical manifestations of LF are primarily due to the tissue localization of long-lived (up to 17 years) adult parasites. Infection by any one of several species of these lymphatic dwelling parasites (*Wuchereria bancrofti*, *Brugia malayi* or *Brugia timori*) is characterized by recurrent fevers, painful adenolymphangitis, lymphoedema (elephantiasis), as well as urogenital swelling and scrotal hydrocele in men. Although not generally considered fatal, filarial infections cause much disfigurement and morbidity, resulting in social stigma with severe economic consequences. An estimated 5.7 million disability-adjusted life years (DALYs) are lost in the diseased population over their lifetime as a result of onchocerciasis [5] and LF infections [6].

Filarial parasites are transmitted by the bite of a blood-sucking arthropod; mosquitoes are the vectors of LF whereas black flies are responsible for spreading onchocerciasis. Mf are ingested by insects while feeding then undergo two molts to become infective third-stage larvae (L3) that are transmitted to the human host during a subsequent bloodmeal. In the human host, larvae molt twice to reach sexual maturity.

Accurate parasite detection is essential for the success of any filariasis control program. In recent years there has been significant progress in the control of filariasis by treating whole populations with repeated, semi-annual or yearly cycles of ivermectin [7]. Traditionally, diagnosis is based on morphological identification of mf in skin biopsies (onchocerciasis) and blood (LF), as well as insect vectors using light microscopy. While morphological interpretation is a valuable technique, it requires substantial expertise, is time consuming and can be subjective. Because mf prevalence decreases in response to mass drug administration (MDA), screening blood pools has become a necessary and cost-effective procedure [8]. However, this method is more likely to produce false-negative results in low mf carriers that may still be infectious to competent insect vectors. Despite these limitations, microscopic detection is still a popular method owing to its low cost and suitability for laboratories with limited resources. The inherent insensitivity of parasitological procedures has prompted the development of several immunological methods involving detection of either antibody or antigen for *W. bancrofti [9, 10], B. malayi* [11–13] and *O*. *volvulus* [14, 15]. However, low levels of sensitivity [16] and cross-reactivity [11, 14, 17, 18] have been reported. Determining the infection levels in vector populations is also important for assessing transmission and deciding when drug treatments may be stopped as well as for monitoring recrudescence [19]. Surveillance involves the capture and dissection of vector insects, followed by microscopic examination of parasites. This method is tedious and requires considerable expertise to distinguish the various filarial nematodes that may exist in an insect.

Nucleic acid-based molecular assays offer higher sensitivity than parasitological or immunological methods and can be used to detect infection in both humans and vectors, as well as to monitor development of drug resistant parasite strains [20]. Polymerase chain reaction (PCR)-based methods have been used for more than 30 years in research laboratories, however the requirement for trained personnel and relatively expensive equipment limit their suitability for field use [21, 22]. More recently, isothermal amplification methods have been developed and are particularly useful for low-resource settings [1, 21] [22]. Loop-mediated isothermal amplification (LAMP) has become the most widely adopted of these methods. LAMP, a single step reaction, can amplify a few copies of target to 10^9^ copies in less than one hour even when large amounts of non-target DNA are present [21]. In addition, the *Bst* DNA polymerases used in LAMP are more tolerant to inhibitors commonly found in clinical specimens and insects which can thwart PCR [23–25]. Determination of amplification is based on simple visual detection of turbidity produced by the precipitation of magnesium pyrophosphate [26]; fluorescence via an intercalating dye [27]; or through a color change of metal-sensitive indicators [28, 29]. This lack of post-amplification processing offers a considerable advantage over PCR [22]. LAMP assays displaying high levels of specificity and sensitivity have been described for various filarial nematodes including *B*. *malayi* [30], *Loa loa* [31, 32], *O*. *volvulus* [25] and *W*. *bancrofti* [33]. These assays can be performed using a simple electric device such as a heat block or water bath set at a single constant temperature. More recently, LAMP assays using a non-instrumented nucleic acid amplification (NINA) heater have been described which greatly facilitate rapid and simple pathogen detection in rural settings [34–37]. Continued improvements to LAMP also include the use of pH sensitive dyes for improved visual detection of the amplification product based on a rapid, distinct and robust color change [38].

In this study, we developed colorimetric NINA-LAMP assays to detect the filarial parasites *B*. *malayi*, *O*. *volvulus and W*. *bancrofti*. We evaluated the efficacy of several strand displacing DNA polymerases in combination with the two pH sensitive dyes, neutral red and phenol red. Conditions for amplification and visualization of a positive result were optimized using purified DNA isolated from each filarial species. Optimized assays were then evaluated further using clinical samples (*W*. *bancrofti* infected blood) or infected insects (*O*. *volvulus* infected black flies or *B*. *malayi* infected mosquitoes).

## Material and methods

### Reagents

DNA and insect samples were generously donated by the following: *B*. *malayi*, L.A. McReynolds (New England Biolabs); *L*. *Loa*, B.L. Makepeace and C. Hartley (University of Liverpool); *W*. *bancrofti*, M. Y. Osei-Atweneboana (Water Research Institute, Accra, Ghana); *O*. *volvulus* infected *Simulium squamosum* black flies were obtained as previously described [39]; uninfected female *Simulium vittatum* black flies were obtained from the Black fly Rearing and Bioassay Laboratory (University of Georgia). *B*. *malayi* infected *Aedes albopictus* adult mosquitoes as well as non-infected adults were obtained from TRS Laboratories (Athens, Georgia). DNA was purified from individual mosquitoes and black flies using a DNeasy Blood and Tissue kit (Qiagen) as instructed by the manufacturer. Whole genome amplified *W*. *bancrofti* DNA was obtained from the NIH/NIAID Filariasis Research Reagent Resource Center (http://www.filariasiscenter.org). Homo sapiens genomic DNA was purchased (Promega, G3041). DNA quantity was determined using a Qubit dsDNA HS Assay kit in conjunction with a Qubit 2.0 Fluorometer as directed by the manufacturer (Life Technologies).

### Colorimetric LAMP assays

LAMP reactions containing neutral red (Sigma-Aldrich) or phenol red (Sigma-Aldrich) dye were set up as described previously [38]. The nucleotide sequences of the *Brugia Hha* I (*BmHha* I), *W*. *bancrofti* Long DNA repeat (*WbLDR*) and *O*. *volvulus* glutathione *S*-transferase 1a *(OvGst1a)* LAMP primer sets used in this study are shown in Table 1. Two loop primers were manually designed to accelerate *W*. *bancrofti* LAMP reactions [33, 40] that were not included in the published primer set (Table 1). Briefly, LAMP reactions contained 1.6 μM each of primers FIP and BIP, 0.2 μM each of F3 and B3, 0.4 μM each of LF and FB, 10 mM (NH4)2SO4, 8 mM MgSO4, 1.4 mM of each dNTP, 0.1% v/v Tween-20, 0.1 mM indicator dye, 8U DNA polymerase (New England Biolabs), and 10 mM KCl if using *Bst* DNA Polymerase, Large fragment (wt*Bst*, LF) or 50 mM KCl, if using *Bst* 2.0 DNA polymerase (*Bst* 2.0) or *Bst* 2.0 WarmStart DNA Polymerase (*Bst* 2.0 WS). Reactions contained template DNA or H_2_O for non-template controls (NTC), with a total volume of 25 μl and pH adjusted to an initial value of 8.2–8.6 at 25°C. A detailed method for reaction setup can be found in S1 Protocol. Reactions were placed in NINA heaters or a qPCR machine (Bio-Rad CFX96) for amplification. In the NINA heaters, a temperature of 63°C was generated by an exothermic reaction initiated by mixing 6 ml of 0.9% saline with a fuel pouch containing 1.15 gm of Mg-Fe mechanically alloyed powder and buffered thermally using a modified, nominally 65°C phase change material. The temperature was monitored inside mock reaction tubes containing 25 μl of H_2_O using type T thermocouples and National Instruments SignalExpress data logging software (www.ni.com) [35]. For *BmHha I* and *WbLDR*, LAMP reactions were placed in the preheated NINA heater optimized to hold samples at 63°C (approximately 15 min after initiation of the exothermic reaction) then incubated for 40 min. *OvGST1a* reactions were added to NINA heaters immediately upon activation with saline and incubated for 70 minutes. When a qPCR machine was used for amplification, SYTO 9 (ThermoFisher Scientific) was added (2 μM final concentration) and reactions were incubated at 63°C for ~ 1h (160 cycles with a plate read step every 15 seconds). To record color changes, samples were scanned using an Epson Perfection v700 photo flatbed scanner.

**Table 1:** Sequences of the *BmHha* I, *OvGST1a* and *WbLDR* LAMP primers.

^a^^,^^b^*BmHha* primers:	Sequence (5’-3’)
FIP	GCTTTTTTTAGTAGTTTTGGCACTTCTTACATTAGACAAGGAAATTGG
BIP	GAAAYTAATTGACTATGTTACGTGCACAACACAATATACGACCAGC
F3	GCGCATAAATTCATCAGC
B3	GCAAAACTTAATTACAAAAGCG
LF	AATTARAATTAAAATTGATAAAT
LB	ATTGTACCAGT
^a^*OvGST1a* primers:	Sequence (5’-3’)
FIP	AATGTTACAGGTAAAGAAGGCATCTTTTGGATATAAACGATGATTTTTCC
BIP	ATCAAGCATAAATGGCCTATTAGCGATGAAACAAATTATAGCGCAAAG
F3	CTCAAAATTACAATTTATCTCTTC
B3	TTTGCCAATGAATGGATT
LF	ATGAAAGAATTCTATTTTAT
LB	GCAAAAATAGAAATGCAT
^a^*WbLDR* primers:	Sequence (5’-3’)
FIP	CGACTGTCTAATCCATTCAGAGTGTATCTGCCCATAGAAATAACTACG
BIP	TCTGTGCTGAATTTTTGTGGATTGCCAAACTAATTGTAAGCAGTCTT
F3	TTTGATCATCTGGGAACGT
B3	AAGCACCTTAAATCTGTCAAT
LF	ATAACCAGAGATCCAC
LB	GTGACGACAACTAGG

^a^The primer sets used to target the *Brugia Hha* I repeat and *O*. *volvulus GST1a* were described previously [25, 41]. Loop primers (LF, LB) were added to the original *WbLDR* primer set [33] to increase amplification speed.

^b^In the *BmHha* I primer set, the degenerate nucleotide Y = C or T and R = A or G.

## Results and discussion

**Fig 1:**
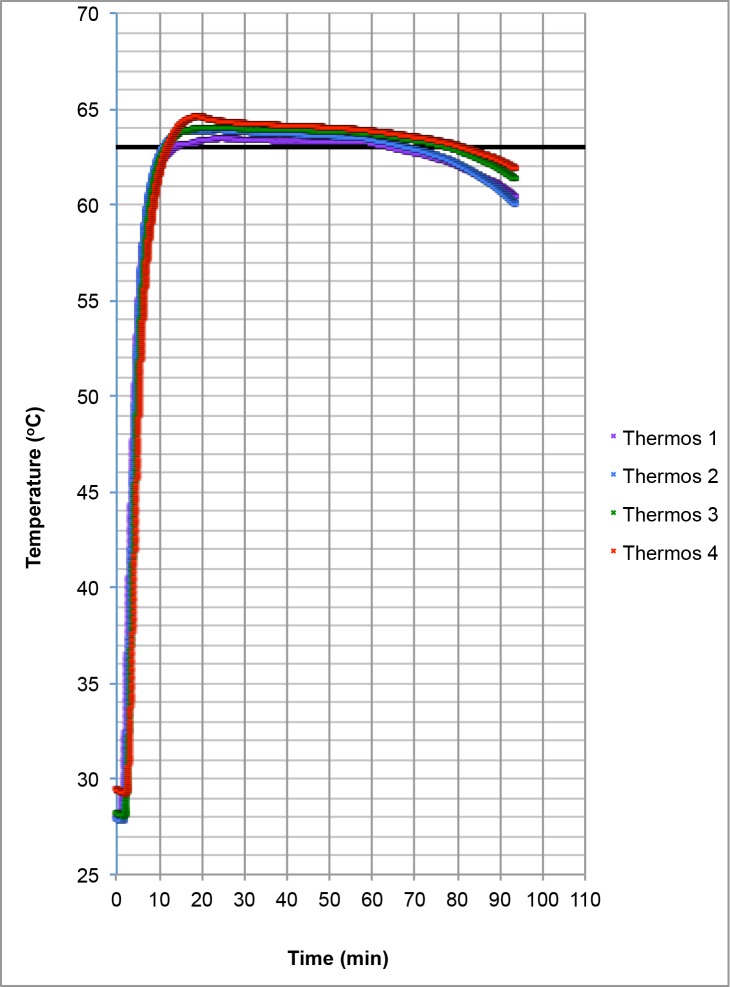
Temperature profile of the NINA heater. The temperature of 25 μl (LAMP reaction volume) of water in PCR tubes containing a T type thermocouple was monitored in four separate heaters using NI Signal Express software. The target temperature of 63°C is denoted by a black line.

The goal of this study was to develop the capacity to diagnose multiple filarial infections of humans and insects simultaneously in a portable electricity-free device (NINA) using a robust and simple colorimetric LAMP assay. Previously published DNA biomarkers were used to detect *B*. *malayi* (*Hha I* repeat, [30]), *O*. *volvulus* (*OvGST1a*, [25]) and W. bancrofti (*WbLDT*, [33]) DNAs. A target temperature of 63°C, ideally suited for the various filarial LAMP assays (62–63°C, [25, 30, 33]), was chosen to develop universal conditions for detection of the three filarial infections using LAMP in the NINA heater [35]. The NINA H.V9 heater is a thermally insulated incubation device designed for isothermal amplification methods. This version is designed to operate in a laboratory setting within an ambient temperature range of 20–25°C although other versions have been designed for broader, uncontrolled ambient temperature ranges. Heat is generated by an exothermic reaction initiated by mixing saline with a Mg-Fe mechanical alloy. The primary exothermic reaction results from the oxidation of Mg with H_2_O, producing MgO, Mg(OH)_2_, and H_2_(g). A secondary galvanic corrosion reaction between the Mg-Fe and saline drives the breakdown of the particles thus maintaining the momentum of the reaction by preventing the buildup of MgO on the accessible surface area of the particles [42]. A phase change material is used to buffer the exothermic reaction providing a temperature range of 62–64°C to samples [37]. To observe the heating dynamics within NINA, the temperature of mock samples containing 25 μl of water was monitored in four heaters with type T thermocouples and SignalExpress data logging software. On average it took approximately 10 minutes for the temperature inside the samples to reach 62°C and approximately 12 minutes to reach 63°C. The highest temperature observed in the samples varied between 63.4–64.3°C. On average, samples maintained a temperature of ≥ 62°C for 76 min and a temperature of ≥ 63°C for 61 min (Fig 1).

**Fig 2:**
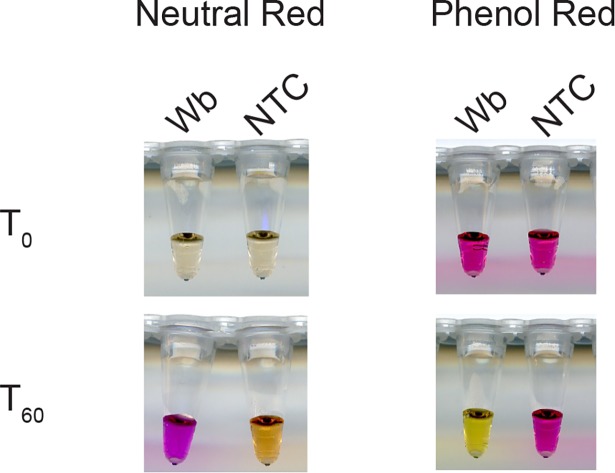
Color change of neutral red and phenol red indicator dyes in LAMP reactions. The *WbLDR* primer set was used to amplify genomic *W*. *bancrofti* (Wb) DNA using the colorimetric master mix containing either neutral red or phenol red dye and *Bst* 2.0 WS. Before amplification (T_0_), reactions containing neutral red are colorless. Samples turn pink if positive or a brownish yellow if negative as shown after a sixty minute (T_60_) amplification. Reactions containing phenol red are pink at T_0_ and remain pink if negative but turn yellow if positive as shown here after a sixty minute (T_60_) amplification.

The temperature profile indicated that the NINA device is suited for the development of colorimetric filarial assays based on published LAMP conditions [25, 30, 33]. Using *W*. *bancrofti* DNA as template, two pH sensitive indicator dyes, neutral red and phenol red were evaluated for colorimetric assay development in NINA-LAMP [38]. Reactions containing neutral red changed from colorless before amplification to pink when positive, or to a brownish yellow in the no template control, whereas reactions containing phenol red were pink initially and remained pink if negative but turned yellow in the presence of template DNA (Fig 2). Both neutral and phenol red provide a clear distinction between positive and negative samples unlike many of the metal-sensitive indicators that can be difficult to distinguish by eye [38].

**Fig 3:**
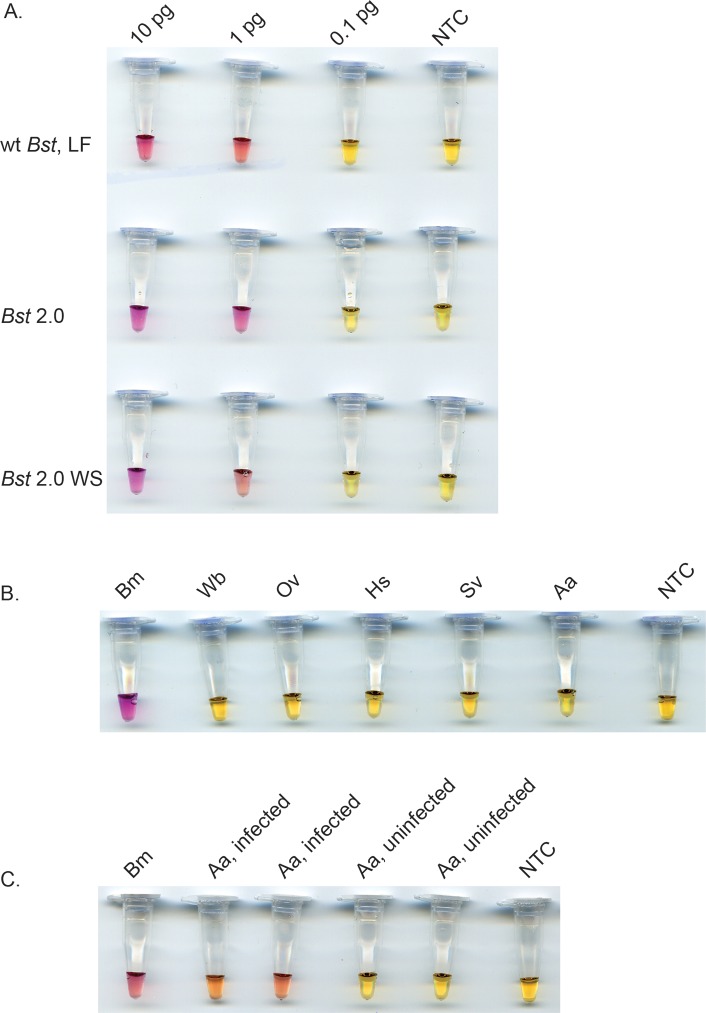
Sensitivity and specificity of the *BmHha* I colorimetric NINA-LAMP assay with neutral red dye. Comparison of sensitivity using *Bst* DNA polymerase, Large Fragment (wt *Bst* LF), *Bst* 2.0 DNA polymerase (*Bst* 2.0) or *Bst* 2.0 WarmStart DNA polymerase (*Bst* 2.0 WS) on a 10X serial dilution of genomic *B*. *malayi* DNA (A). Species-specificity using 100 pg of various genomic DNAs (B). Detection of *B*. *malayi* DNA in mosquitoes (C). Species names are abbreviated as follows: *B*. *malayi* (Bm), *W*. *bancrofti* (Wb), *O*. *volvulus* (Ov), *Homo sapiens* (Hs), *Simulium vittatum* (Sv) and *Aedes aegypti* (Aa). The non-template controls (NTCs) contain water.

To determine the most suitable DNA polymerase for use, several strand displacing polymerases, wt *Bst* LF, *Bst* 2.0 and *Bst* 2.0 WS were evaluated in the *B*. *malayi* colorimetric NINA-LAMP platform (Fig 3). *Bst* 2.0 is a recently engineered enzyme that provides faster reaction speed and increased tolerance to salt and other impurities. *Bst* 2.0 WS further allows setup of reactions at ambient temperature due to the presence of a temperature sensitive aptamer. All three DNA polymerases reliably detected 1.0 pg (1/100^th^ of mf) of genomic *B*. *malayi* DNA in 40 minutes as determined by a color change to pink using neutral red (Fig 3A). Negative reactions turned yellow as seen for the samples containing 0.1 pg *B*. *malayi* DNA or water. Increasing the assay time to 60 minutes did not improve sensitivity (data not shown). While sensitivity of detection was consistent between DNA polymerases, the intensity of the color observed varied slightly perhaps due to the different potassium chloride concentrations in the master mix or alternatively, the overall level of amplification that occurred in a particular sample. Given that no differences in sensitivity were observed between the three strand displacing polymerases tested, *Bst* 2.0 WS was chosen for amplification of each filarial DNA in all subsequent experiments since its warm start characteristics enable simplified reaction set up at ambient temperatures. We found the sensitivity of *Brugia* colorimetric NINA-LAMP to be comparable to that reported for standard LAMP [30] and PCR [43].

Specificity of the *B*. *malayi* colorimetric NINA-LAMP assay was also evaluated and amplification was only observed in the presence of DNA from *B*. *malayi*. Reactions containing genomic *B*. *malayi* DNA scored positive (pink) whereas those containing DNA from the closely related *W*. *bancrofti* and *O*. *volvulus parasites*, or from human, black fly or mosquito, were negative (yellow) (Fig 3B). The LAMP mechanism which employs 4–6 primers that hybridize to 6–8 regions in the target generates exquisite specificity [21, 22, 40] offering a distinct advantage over the antibody detection assays for *Brugia* diagnosis [12] [13] which exhibit cross-reactivity [11, 44] with other filarial nematodes thus limiting their utility in regions where these infections co-exist. The lack of reactivity with mosquito DNA, enabled us to explore the possibility of using the *B*. *malayi* colorimetric NINA-LAMP assay for insect surveillance. DNA was isolated from both infected and non-infected mosquitoes and examined. Only samples from infected mosquitoes scored positive (pink), whereas samples containing DNA from non-infected mosquitoes or water scored negative (yellow) (Fig 3C). Recent studies suggest that LAMP may be more sensitive than PCR for quantification of infection rates among mosquitoes [45] further supporting the use of the colorimetric NINA-LAMP method as a tool for monitoring transmission.

A colorimetric NINA-LAMP assay for *O*. *volvulus* based on a recently described biomarker, OvGst1a, was also developed. Standard *OvGST1a*-based LAMP and PCR assays are capable of differentiating *O*. *volvulus* from *O*. *ochengi*, a filarial parasite of cattle in West Africa. Both species are transmitted by the *Simulium damnosum sensu lato* complex of black fly vectors [46]. Accurate data about *O*. *volvulus* infection rates in black flies depends on the ability to differentiate *O*. *volvulus* from *O*. *ochengi* in the vector population. The standard nucleic acid target employed for detection of *Onchocerca* is O-150, a genus specific repeat family [47, 48]. PCR differentiation between O-150 repeats from *O*. *volvulus* and *O*. *chengi* requires additional hybridization steps with a specific *O*. *volvulus* DNA probe to achieve specificity [49] whereas OvGST1a can be used in a single step reaction without geographical restriction [25].

**Fig 4:**
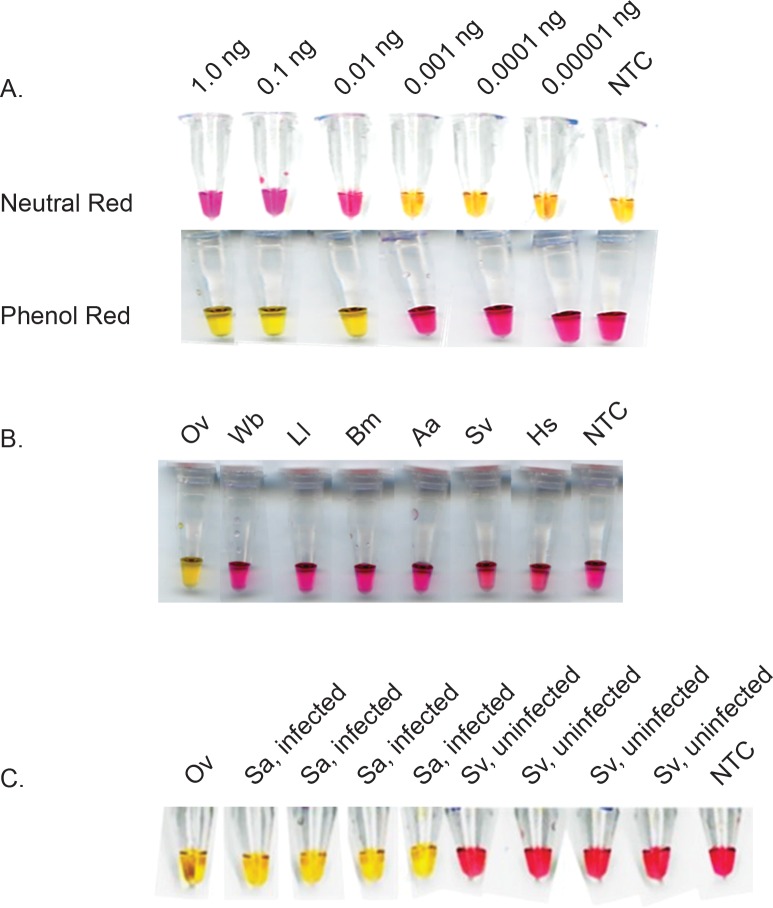
Sensitivity and specificity of the *OvGST*1a colorimetric NINA-LAMP assay using *Bst* 2.0 WS. Sensitivity comparison of neutral red and phenol red dyes on a 10X dilution series of genomic *O*. *volvulus* DNA (A). Species-specificity using 1 ng of various genomic DNAs (B). Detection of *O*. *volvulus* in black flies (C). Species names are abbreviated as follows: *O*. *volvulus* (Ov); *W*. *bancrofti* (Wb); *Loa loa* (Ll); *B*. *malayi* (Bm); *Aedes aegypti* (Aa); *Simulium vittatum* (Sv); *Simulium squamosum* (Sa) and *Homo sapiens* (Hs). NTC = Non-template control.

Sensitivity of colorimetric NINA-LAMP was determined using samples containing 0.00001–1.0 ng of genomic *O*. *volvulus* DNA in the presence of either neutral red or phenol red dyes. Reactions containing neutral red turned pink when positive or brownish yellow if negative whereas reactions containing phenol red turned yellow if positive and but remained pink when negative (Figs 2 and 4A). Irrespective of the dye used, 0.01 ng (1/10^th^ of mf) of *O*. *volvulus* DNA was detected using NINA-LAMP (Fig 4A). Reactions containing 0.001 ng of genomic *O*. *volvulus* DNA or less including the NTCs were negative. This is consistent with the level of sensitivity previously reported using standard LAMP conditions and turbidity or hydroxy napthol blue as the readout or by PCR [25]. No difference in sensitivity was observed between neutral red or phenol red when used in the colorimetric NINA-LAMP tests for either brugian or bancroftian filariasis (data not shown).

The specificity of the *O*. *volvulus* colorimetric NINA-LAMP assay was evaluated using phenol red with *Bst* 2.0 WS and 1 ng of various genomic DNAs. The assay successfully differentiated samples containing *O*. *volvulus* (by turning yellow) from those containing closely related filarial DNAs or DNA from human or black fly vector (remained pink) (Fig 4B).

To further evaluate the suitability of the NINA device for use in field conditions, DNA isolated from experimentally infected *Simulium squamosum* black flies was assayed (Fig 4C). All four of the DNA samples purified from the individually infected flies as well as the *O*. *volvulus* genomic DNA control scored positive (yellow) whereas the DNA samples purified from non-infected flies as well as the NTC scored negative (pink) (Fig 4C). This assay is also likely to be useful in analyzing pools of infected black flies as we have previously shown high levels of sensitivity in standard LAMP assays using DNA samples prepared from pools containing 200 black flies spiked with 0.1 ng *O*. *volvulus* DNA [25]. These results highlight the potential of the *O*. *volvulus* colorimetric NINA-LAMP platform as a surveillance tool for *O*. *volvulus*-infected vectors in endemic communities.

**Fig 5:**
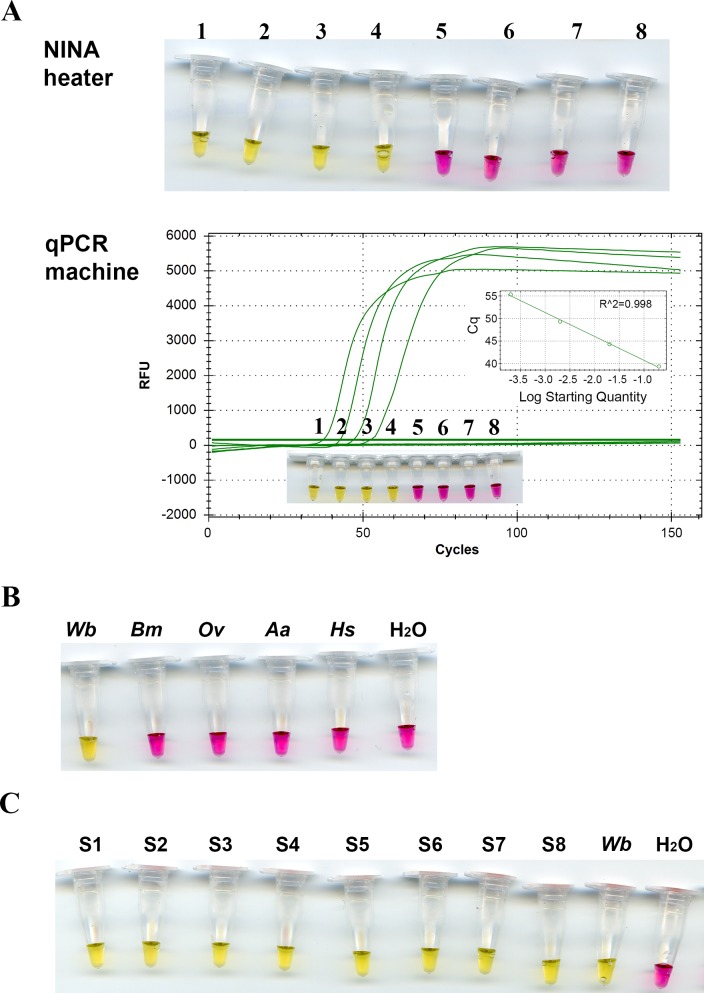
Sensitivity and specificity of the *WbLDR* colorimetric NINA-LAMP assay using *Bst* 2.0 WS. (A) LAMP reactions incubated in the NINA heater exhibit the same sensitivity as those incubated in a qPCR machine with DNA amplification monitored in real time using a dsDNA binding dye. Genomic *W*. *bancrofti* DNA dilutions equivalent to 1/5 mf (1), 1/50 mf (2), 1/500 mf (3), 1/5000 mf (4), 1/50,000 mf (5), 1/500,000 mf (6), 1/5,000,000 mf (7) and H_2_O (8) were used as template. The end point color change of the LAMP reactions incubated in the NINA heater are shown in the upper panel. Real time amplification curves and end point color change of LAMP reactions are shown in the lower panel. (B) Species-specificity using 100 pg of various genomic DNAs as template. (C) Detection of *W*. *bancrofti* DNA in blood samples from 8 different LF positive patients (S1-S8). Species names are abbreviated as follows: *W*. *bancrofti* (*Wb*), *B*. *malayi* (*Bm*), *O*. *volvulus* (*Ov*), *Aedes aegypti* (*Aa*) and *Homo sapiens* (*Hs*). All experiments were performed in triplicate. One representative of each is shown.

A colorimetric NINA-LAMP assay was developed for *W*. *bancrofti* using previously published LAMP primer set targeting the nuclear scaffold/matrix attachment region (GenBank accession no. AY297458) also known as the *W*. *bancrofti* Long DNA repeat (*WbLDR*) [1, 50]. The limit of sensitivity of this assay is reported to be approximately 1/1000^th^ of an mf or 0.1 pg of genomic *W*. *bancrofti* DNA [33]. To increase assay speed, two additional loop primers (Table 1) were designed and included. Ten-fold serially diluted DNA extracted from 200 *W*. *bancrofti* mf was used as template for colorimetric LAMP reactions containing phenol red and *Bst* 2.0 WS to determine sensitivity (Fig 5A). DNA from 1/5000th of an mf scored positive (yellow), representing a 5-fold increase in sensitivity compared to the previously published LAMP assay [33]. A duplicate set of samples containing SYTO 9 were amplified in a qPCR machine set at 63°C to monitor the dynamics of the colorimetric LAMP reaction. Analysis of the real-time amplification signal from the positive LAMP reactions corresponded to the end point color change in the same samples. Furthermore, a good linear correlation of reaction speed with log amount of DNA added to the reactions was observed suggesting that the assay is semi-quantitative and can be used to estimate infection load (Fig 5A). This semi-quantitative quality of LAMP has been reported elsewhere [51]. Concordant results were obtained using both the qPCR and NINA devices, confirming the reliability of the NINA heater and the end point color change.

The specificity of the *W*. *bancrofti* NINA-LAMP assay was evaluated using phenol red with *Bst* 2.0 WS and 100 pg of various genomic DNAs (Fig 5B). The assay successfully differentiated the sample containing *W*. *bancrofti DNA* (by turning yellow) from samples containing closely related filarial parasites as well as human and vector DNA samples (remained pink) (Fig 5B). These results are consistent with the specificity of the previously published *WbLDR* primer set [33] using standard LAMP conditions demonstrating that the addition of loop primers did not compromise assay specificity. With its enhanced sensitivity and high level of specificity, the *W*. *bancrofti* colorimetric NINA-LAMP assay may prove a suitable alternative to the circulating female antigen assay (CFA) (Alere Filariasis Test Strip) commonly used to diagnose *W*. *bancrofti* infection that cannot be employed in areas where *Loa loa* is endemic due to issues with cross-reactivity [18, 52–54].

To evaluate whether the colorimetric NINA-LAMP platform might be suitable for detecting infected blood samples under field conditions, DNA extracted from 8 different *W*. *bancrofti* samples of mf was assayed using *W*. *bancrofti* colorimetric NINA-LAMP (Fig 5C). All eight DNA samples and the control scored positive (yellow) whereas the non-template water control scored negative (pink) (Fig 5C). These results suggest that the enhanced sensitivity of the *W*. *bancrofti* colorimetric NINA-LAMP assay may be suitable for detecting infection in humans. In addition, questions have arisen regarding the sensitivity of the *W*. *bancrofti* CFA assay in regions where the endemicity of *W*. *bancrofti* is low or that have undergone multiple rounds of MDA [16]. These issues have prompted the use of the *WbLDR* LAMP assay for monitoring mosquitoes as a surrogate for the CFA test in low endemic regions [45]. Therefore, the *W*. *bancrofti* colorimetric NINA-LAMP may be suited for both diagnosis of infection in humans and surveillance of insect vectors.

In summary, nucleic acid based approaches to detect filarial infection offer considerable advantages over traditional parasitological and immunological methods. They offer greater levels of sensitivity and specificity, and versatility as the same test can be used for human and insect hosts [1]. In recent years there have been many advances resulting in the availability of cheaper and simpler molecular diagnostic tests suitable for low resource settings. We describe the use of a single portable electricity free device (NINA) to perform colorimetric LAMP reactions for the detection of several filarial species present in human samples or insect vectors. The assay is easily adapted to accommodate multiple different primer/target combinations. Reaction times varied depending on whether a single copy gene (70 minutes, *O*. *volvulus*) or a repetitive DNA target (40 minutes, *B*. *malayi* and *W*. *bancrofti*) was used. The simplicity and versatility of the technology indicates that the colorimetric NINA-LAMP filarial assays are ideally suited for monitoring the success of MDA programs.

## Supporting information

S1 Protocol Colorimetric LAMP Protocol for the detection of *BmHha* I, *OvGST1a* and *WbLDT*. (DOCX)
