# Supplementary material for: Colorimetric tests for diagnosis of filarial infection and vector surveillance using non-instrumented nucleic acid loop-mediated isothermal amplification (NINA-LAMP)
Source: PLoS One. 2017 Feb 15;12(2):e0169011. doi: 10.1371/journal.pone.0169011 (PMC5310896; doi:10.1371/journal.pone.0169011)
Supplement: S1 Protocol — (DOCX) [file pone.0169011.s001.docx]

**Colorimetric LAMP Protocol for the detection of *BmHha* I, *OvGST1a* and *WbLDT***

| Components | Volume (μl) | 2X concentration | 1X concentration |
| --- | --- | --- | --- |
| 10 mM dNTP solution mix (NEB# N0447) | 1400 | 2.8 mM | 1.4 mM |
| 3M (NH_4_)_2_SO_4_ | 33.4 | 20 mM | 10 mM |
| 100 mM MgSO_4_ | 800 | 16 mM | 8 mM |
| ^a^2M KCl | 50 (if wt*Bst,* LF) | 20 mM | 10 mM |
|  | 250 (if *Bst* 2.0 or *Bst* 2.0 WS) | 100 mM | 50 mM |
| Tween 20 | 10 | 0.2 % v/v | 0.1 % v/v |
| ^b^H_2_O | 2165.3 (if wt*Bst*, LF) | --- | --- |
|  | 2005.3 (if *Bst* 2.0 or *Bst* 2.0 WS) | --- | --- |
| ^c^Total Volume | 5000 | --- | --- |

**1.** **Prepare** **2X colorimetric solution mix:**

**2. Prepare 25X Dye Stocks of neutral red or phenol red:**

| Components | Volume (μl) | 25X concentration |
| --- | --- | --- |
| ^d^50 mM Dye solution | 50 | 2.5 mM |
| H_2_O | 950 | --- |
| ^e^Total Volume | 1000 | --- |

**3. ^c^Master Mix:**

| Components | Volume (μl)/rxn | Volume (μl)/100 rxn |
| --- | --- | --- |
| 2X colorimetric solution mix pH 8.6-8.8 | 12.5 | 1250 |
| 25X Dye solution pH 8.6 | 1 | 100 |
| ^f^*Bst* polymerase (120,000 U/ml) | 0.067 | 6.7 |
| ^b^H_2_O | 1.433 | 143.3 |

**4. 25X Primer Mixes:**

| ^g^Standard Primers | Volume (μl) | 25X concentration | 1X concenration |  |
| --- | --- | --- | --- | --- |
| 100 μM FIP | 40 | 40 μM | 1.6 μM |  |
| 100 μM F3 | 5 | 5 μM | 0.2 μM |  |
| 100 μM BIP | 40 | 40 μM | 1.6 μM |  |
| 100 μM B3 | 5 | 5 μM | 0.2 μM |  |
| H_2_O | 10 | ---- | ---- |  |
| Total Volume | 100 | ---- | ---- |  |

| ^g^Loop Primers | Volume (μl) | 25X concentration | 1X concentration |  |
| --- | --- | --- | --- | --- |
| 100 μM LF | 10 | 10 μM | 0.4 μM |  |
| 100 μM LB | 10 | 10 μM | 0.4 μM |  |
| H_2_O | 80 | ---- | ---- |  |
| Total Volume | 100 | ---- | ---- |  |

**5. ^h^Colorimetric LAMP reactions:**

| Components | Volume (μl) |
| --- | --- |
| Master mix pH 8.6-8.8 | 15 |
| 25X Standard Primer mix | 1 |
| 25X Loop primer mix | 1 |
| ^i^Substrate DNA | 2 |
| H_2_O | 6 |
| Total Volume | 25 |

a. Separate solution mixes must be prepared for *Bst* DNA polymerase, Large fragment (wt*Bst,* LF) and *Bst* DNA polymerase 2.0 (*Bst* 2.0) as well as its WarmStart version (*Bst* 2.0 WS) as these enzymes require different KCl concentrations.

b. Adjust the pH of the solution to between 8.6-8.8 with 1M KOH before bringing the solution up to its final volume with H_2_O.

c. Store aliquots frozen in screw top tubes.

d. Neutral red and phenol red dyes are available from Sigma-Aldrich.

e. Adjust the pH of the dye solution to 8.6 or greater with 1M KOH before bringing the solution up to its final volume with H_2_O.

f. Highly concentrated polymerase is used to minimize carry over of Tris into the master mix. NEB catalog numbers for concentrated polymerase are as follows: wt*Bst* LF, M0275M; *Bst* 2.0, M0537M and *Bst* 2.0 WS, M0538M.

g. 100 μM primer stocks are prepared in H_2_O to minimize carry over of Tris.

h. LAMP reactions are incubated in a NINA heater @ 63^o^C for 40 to 70 min depending on the primer set as described in the Materials and Methods.

i. Substrate DNA can be dissolved in either T_10_E _0.1_ or H_2_O.
